# Docosahexaenoic Acid Counteracts the Hypoxic-Induced Inflammatory and Metabolic Alterations in 3T3-L1 Adipocytes

**DOI:** 10.3390/nu14214600

**Published:** 2022-11-01

**Authors:** Noura B. Younes, Omnia Ahmed Mohamed, Nasser M. Rizk

**Affiliations:** 1 Biomedical Sciences Department, College of Health Sciences, QU Health, Qatar University, Doha 2713, Qatar; 2 Clinical Chemistry Laboratory, Hamad Medical Corporation, Doha 2713, Qatar; 3 Biomedical Research Center (BRC), Qatar University, Doha 2713, Qatar

**Keywords:** docosahexaenoic acid (DHA), obesity, hypoxia, 3T3-L1 cell adipocytes, adipokines, metabolism

## Abstract

Background: Hypoxia is caused by the excessive expansion of the white adipose tissue (AT) and is associated with obesity-related conditions such as insulin resistance, inflammation, and oxidative stress. Docosahexaenoic acid (DHA) is an omega-3 fatty acid reported to have beneficial health effects. However, the effects of DHA in AT against hypoxia-induced immune-metabolic perturbations in adipocytes exposed to low O_2_ tension are not well known. Consequently, this study aimed to evaluate the impact of DHA on markers of inflammation, metabolism, apoptosis, and oxidative stress in 3T3-L1 cell adipocytes exposed to low O_2_ tension (1% O_2_) induced hypoxia. Methods: The apoptosis and reactive oxygen species (ROS) rates were evaluated. Metabolic parameters such as lactate, FFA, glycerol release, glucose uptake, and ATP content were assessed by a fluorometer. The expression of HIF-1, GLUT1 and the secretion of adipocytokines such as leptin, adiponectin, and pro-inflammatory markers was evaluated. Results: DHA-treated hypoxic cells showed significantly decreased basal free fatty acid release, lactate production, and enhanced glucose consumption. In addition, DHA-treatment of hypoxic cells caused a significant reduction in the apoptosis rate and ROS production with decreased lipid peroxidation. Moreover, DHA-treatment of hypoxic cells caused a decreased secretion of pro-inflammatory markers (IL-6, MCP-1) and leptin and increased adiponectin secretion compared with hypoxic cells. Furthermore, DHA-treatment of hypoxic cells caused significant reductions in the expression of genes related to hypoxia (HIF-1, HIF-2), anaerobic metabolism (GLUT1 and Ldha), ATP production (ANT2), and fat metabolism (FASN and PPARY). Conclusion: This study suggests that DHA can exert potential anti-obesity effects by reducing the secretion of inflammatory adipokines, oxidative stress, lipolysis, and apoptosis.

## 1. Introduction

Adipose tissue (AT) is an endocrine organ that discharges fatty acids, proteins, and peptides named adipokines. The adipocytes produce more than sixty adipokines as signaling molecules by which the adipose tissue can communicate with other tissues and organs. These adipokines are engaged in several biological processes, such as regulating energy balance, controlling glucose and lipid metabolism, insulin-sensitizing action, immune system responses, inflammation, hemostasis, and angiogenesis [1]. Specific adipokines are affiliated with the distinctive stigma of AT hypertrophy, characterized by chronic low-grade inflammation, and are interconnected with the pathogenesis of metabolic disorders related to obesity [1]. Hypertrophic obesity is characterized by the increased adipocyte size, which could reach up to 140–180 μm in diameter [2], which exceeds the normal distance for O_2_ diffusion, i.e., 100–200 μm [3]. Consequently, hypertrophic adipocytes are distressed because of inadequate oxygen supply, and hypoxia develops.

Hypoxia-inducible factor 1 (HIF-1) activation maintains adipocytes’ cellular compliance to hypoxia. During hypoxia, HIF-1 increases and upregulates the mRNA expression of numerous genes that stimulate angiogenesis, glycolysis, and erythropoiesis [4]. Hypoxia has numerous impacts on the cellular level, such as the production of reactive oxygen species (ROS), the increase in the expression of various pro-inflammatory adipocytokines, and the decreased expression of anti-inflammatory factors, including IL-10 and adiponectin [5]. HIF-1 is also implicated in hypoxia-induced insulin resistance and switches the cell metabolism from aerobic to anaerobic with a decrease in ATP output and an increase in lactic acid generation [6]. Adipose tissue hypertrophy with low-grade inflammation caused by hypoxia has been established in clinical [6] and experimental studies [7]; thus, alternative strategies to improve cellular hypoxia are sought as a kind of anti-obesity treatment.

Omega-3 long-chain PUFAs, such as docosahexaenoic acid (DHA), also known as C22:6 n-3 docosahexaenoic acid, have attracted the attention of several research institutes all over the world due to their positive effects on human health [8]. These dietary fats are integrated into various regions of the body, including the cell membranes. They participate in anti-inflammatory processes and have a wide range of positive effects on health [9]. DHA is also a precursor of several potent lipid mediators that are beneficial in preventing or treating several diseases [10]. In addition, omega-3 fatty acids have recently drawn much attention as an anti-obesity medication for humans [11].

Previous reports, including experimental as well as clinical studies, demonstrated that dietary supplementation with fish oil, which contains a high concentration of long-chain omega-3 polyunsaturated fatty acids (omega-3 FA), such as EPA and DHA, has beneficial effects against obesity and its comorbidities [11,12,13,14,15]. Little is known about the effect of DHA in protecting AT against hypoxia-induced immune-metabolic perturbations in obesity. A few clinical studies demonstrated that long-chain omega-3 polyunsaturated fatty acids for 12 weeks [16] and a food supplement consisting of eicosatetraenoic acid (EPA) and docosahexaenoic acid (DHA) [17] could reduce the expression of hypoxia-inducible factor (HIF-1) suggesting a reduction in hypoxia in subcutaneous adipose tissue. Moreover, these studies reported that long-chain omega-3 polyunsaturated fatty acids could modulate the genes involved in obesity development in human subcutaneous adipose tissue, such as downregulation of PPAR gamma and PGC-1alpha and an upregulated expression of the genes encoding PPAR alpha and SREBP1. Therefore, the purpose of the present experimental study is to evaluate the effect of DHA on markers of inflammation, metabolism, apoptosis, and oxidative stress in 3T3-L1 cell adipocytes that were exposed to hypoxia characterized by low O_2_ tension (1% O_2_), which mimics hypertrophic obesity and insulin resistance in humans.

## 2. Materials and Methods

### 2.1. Materials

Cryopreserved 3T3-L1 pre-adipocytes [Cat# SP-L1-F] were purchased from Zen-Bio Company (Chapel Hill-Nelson Blvd., Suite 104, PO Box13888/ Research Triangle Park, NC 27709, USA). The media were purchased alongside the cells from the same company (Zen-Bio). The media were: 3T3-L1Preadipocyte Media, Cat# PM-1-L-1 (contains 4.5 g/L D-glucose, equals ~25 mM); 3T3-L1Adipocyte Maintenance Media, Cat# AM-1-L-1; 3T3-L1 Adipocyte Maintenance basal Media without serum, Cat# AM-1-L-1-DF; 3T3-L1 Adipocyte Differentiation Medium, Cat# DM-2-L-1, see media formulations in “Supplementary Table S1”; and Cryopreservation Medium for 3T3-L1 cells, Cat# FM-1-L-1-100. All media except Preadipocytes Media, Cat# PM-1-L-1, contained 3.15g/L D-glucose (equals ~17.5 mM). Dulbecco’s Modified Eagle Medium (Cat#12491015) was provided by ThermoFischer, Waltham, MA, USA. 3T3-L1 Lipolysis Assay KIT (Cat#LIP-3-NC-L1) Combo-Free Fatty acid and glycerol release were obtained from Zen-Bio Company, Durham, NC, United States. Trypsin/EDTA (TE) solution (1X), REF# R-001-100, was purchased from Gibco (Life Technologies, Inchinnan, Renfrew UK). Phosphate Buffered Saline (PBS) pH 7.4 (1X), #10010023, was purchased from Gibco (Life Technologies, Inchinnan, Renfrew UK). TRIzol Reagent Ambion RNA [REF #15596026] was purchased from Life Technologies Grand Island, NY 14072, USA. Chloroform HPLC grade, C/4966/15, was purchased from Fisher Scientific, Waltham, MA, USA. A High-Capacity RNA-to-cDNA kit was purchased from Applied Biosystems, Foster City, USA, Cat#P/N 4387406. TaqMan Gene Expression Master Mix (Cat#P/N 4369016); pre-developed TaqMan Assay Reagents, Mouse Beta-Actin (20X) (Ref# Mm00607939_s1); TaqMan® Gene Expression Assays, Ref# Mm01184322_m1 (Mouse PPARγ primer); TaqMan® Gene Expression Assays, Ref# Mm00468869_m1 (Mouse Hif 1a primer), and TaqMan® Gene Expression Assays, Ref# Mm00600697_m1 (Mouse GluT-1 primer), TaqMan® Gene Expression Assays, Ref# Mm00662319_m1 (Mouse FASN- mouse primer) were all purchased from Applied Biosystems, Foster City, CA, USA, ThermoFischer, USA. PrimeTime® Mini qPCR Assay, Mm. PT.58.13819524, HIF-2 ALPHA Probe 5′-/56-FAM/ACC AGA GCC/ZEN/GTT TTT GAG AGT CAG G/3IABkFQ/-3′ Primer1 5′-GAC ACG TCT TTG CTC TTC TTC-3′, Ref# 68681817; Primer2 5′-GAC TTC ACT CAT CCT TGC GA-3′; PrimeTime® Mini qPCR Assay, Mm. PT.58.9683859, GLUT 4 Probe, 5′-/56-FAM/TGG AAA CCC/ZEN/GAC GGC ATC TTG T/3IABkFQ/-3′ Primer 1 5′-GAG AATACA GCT AGG ACC AGT G-3′ Primer 2, 5′-TCT TAT TGC AGC GCC TGA G-3′, Ref# 68681821 and PrimeTime® Mini qPCR Assay, Mm. PT.58.32860004, ANT 2 PrimeTime Probe,5′-/56-FAM/TCA CGG CAG/ZEN/ATA AGC AATACA AGG GC/3IABkFQ/-3′ Primer1, 5′-GAT ACG AAC CAC GCA GTC TAT G-3′ Primer 2, 5′-GCA GCC ATC TCC AAG ACA G-3′, Ref# 68681825 were all purchased from IDT Integrated DNA Technologies, Coralville, IA, USA. 

In addition, Adiponectin Mouse ELISA (RD293023100R), Leptin Mouse/Rat Elisa (RD291001200R), Interleukin-6 Mouse ELISA (RAF071R), and Monocyte Chemotactic Protein-1 Mouse ELISA (RAF080R) were all purchased from BioVendor.Inc, International, Asheville, NC, USA. Lipid Peroxidation malondialdehyde (MDA) Assay Kit (ab118970), and ATP Assay Kit (ab83355), were all purchased from Abcam, Cambridge, MA, USA. Docosahexaenoic acid DHA (Substance Name: 4,7,10,13,16,19-Docosahexaenoic acid), Cat. No. U-84-A was purchased from Nu-Chek-Prep., Inc., Elysian, MN, USA. All other study materials were high-quality and purchased from Sigma (St. Louis, MO, USA).

### 2.2. Cell Culture

Cryopreserved 3T3L-1 pre-adipocytes cell passage 14 obtained from Zenbio (ZenBio, Inc. 3, Durham, NC, USA) were used for the present study. First, 3T3-L1 pre-adipocytes were expanded to passage 17, which we used for all the experiments. The differentiation was performed following the ZenBio 3T3-L1 Cell Care Manual, as shown in Supplementary Figure S1, and the media formulations are described in Supplementary data. Following the expansion, 3T3L-1 Cells were seeded and maintained with a Preadipocyte Medium (PM-1-L1), where the pre-adipocytes were incubated until they were 80–85% confluent. Next, 3T3-L1 pre-adipocytes passage 17 were seeded in 6 well plates at a density of 50 × 10^3^ cells per well and maintained until reaching 100% confluence, then incubated at 37 °C and 5% CO_2_ for an extra 48 h. Then, the equal volume of medium was exchanged with the Differentiation Medium (DM) (Day 0) and incubated for 72 h at 37 °C in a humidified atmosphere containing 5% CO_2._ Afterward, the DM was exchanged with Adipocyte Maintenance Medium (AMM); AMM was changed every 48 h. 3T3-L1 Pre-adipocyte cells are completely differentiated between 7 to 10 days after DM application, as evidenced by oil red staining and as previously published [18].

#### 2.2.1. Oil Red O Staining

Using Oil Red O Staining 14, this experiment analyzes the secretory effect of differentiated 3T3-L1 adipocytes 7 to 10 days after DM administration [19]. First, 3T3-L1 adipocytes were rinsed with PBS, then fixed for six minutes with 10% formalin. Following washing with distilled water, the cells were stained for 15 min at 37 °C with oil red O solution, as previously published [19]. Photographs of stained cells were collected using an Olympus DP72 microscope, as shown in the results section. 

#### 2.2.2. Hypoxia and DHA Treatment

In place of hypoxic incubations, the medium was exchanged with Basal Medium DMEM serum-free medium, and the adipocytes normoxic conditions group was incubated in 5% CO_2_ and 95% air. For the hypoxic condition group, the plates were cultured in 5% O_2_ in a gas-impermeable chamber (Xvivo hypoxia chamber system, Biospherix, Parish, NY, USA). The flushed gas mixture consisted of 1% O_2_, 94% N_2_, and 5% CO_2_. For the third group, 3T3-L1 adipocytes were cultured for 24 h in the presence of 50 μM DHA complexed to albumin under hypoxia 1% O_2_ condition. The dose was chosen based on prior studies [20,21], where the dose has no toxicity or effect on cell viability. DHA was delivered to the cells as fatty acid/bovine serum albumin (BSA) complexes. DHA was produced as shown in the supplemental materials. In brief, DHA was dissolved in ethanol to a concentration of 1 mg/mL and then mixed with 8.4% fatty acid-free bovine serum albumin. The fatty acid molar ratio to BSA was 4:1. Prior to each experiment, a fresh solution of the fatty acid-albumin complex was prepared. After a 12-h serum fast, 3T3-L1 adipocytes were treated for 24 h with DHA-BSA stock solutions that had been previously diluted in serum-free growth media (DMEM supplemented with 1% FA-free BSA and 1% antibiotic) with a final concentration of 50 µM of DHA as previously published [22]. 3T3L-1 cells in the control group were cultured in a humidified atmosphere (5% CO_2_, 95% air) to control normoxia at 37 °C. Therefore, we have three groups for study: 

Group 1: 3T3-L1 adipocytes cells with normoxia (CN) 21% O_2_ (24 h) treatment. Group 2: 3T3-L1 adipocytes cells with hypoxia 1% O_2_ (24 h) treatment (Hyp). Group 3: 3T3-L1 adipocytes cells with hypoxia 1% O_2_+DHA (24 h) treatment (Hyp+DHA). As previously published, cell count and viability for different treatment groups were evaluated using a Tail-image-based cytometer [23].

### 2.3. Assessment of Apoptosis 

The percentage of apoptotic cells in the sample was assessed using the TaliTM Apoptosis Kit-AnnexinV Alexa FluorTM 488 and Propidium Iodide and the Tali® Image-Based Cytometer. After 24 h of hypoxia, hypoxia + DHA, and normoxia, 3T3-L1 adipocytes were trypsinized with 3 mL TE to harvest the treated cells. The supernatant was removed after 37 °C incubation for 5–7 min and 500× centrifugation. In a microcentrifuge tube, cells were resuspended in 1X Annexin binding buffer (ABB) and Annexin V Alexa Fluor® 488. Vortexed and incubated for 20 min at room temperature in the dark. After 500× *g* centrifugation, the pellet was resuspended in 100 l of ABB and 1 l of Tali® PI, briefly mixed, and incubated at room temperature for 1–5 min. 25 µL of the stained cells was put onto a Tali® Cellular Analysis slide, and the percentage apoptosis of all samples was assessed using a Tali® Image-Based Cytometer at 488/499 nm and 535/617 nm Ex/Em wavelengths for Annexin V Alexa Fluor® 488 and PI, respectively [23,24]. 

### 2.4. Assessment of Reactive Oxygen Species

3T3-L1 adipocytes of different groups were examined for oxidative stress. Carboxy-H2-DCFDA labeling was used to detect total ROS in live cells according to the manufacturer’s recommendations. Cell-ROX® positive cells were counted using a Tali-image-based cytometer, while H2-DCFDA positive cells were observed using an inverted fluorescent microscope (Olympus X51). Reactive oxygen species were measured using CellROX® orange reagent (Invitrogen) and Tali image-based cytometer 19. First, all treated cells were extracted using 3ml TE, then centrifuged and resuspended in 200 µL of PBS. Next, 5 M (0.4 L) of CellROX® orange reagent was added, and the mixture was briefly vortexed before incubation at 37 °C for 30 min. Next, the cells were separated by centrifugation at 500× *g* and washed three times with PBS. Finally, 25 μL of the labeled cells was put onto the Tali Cellular Analysis slide, and all samples were evaluated using Tali® Image-Based Cytometer at 545/565 nm Ex/Em wavelength. After hypoxia, normoxia, and DHA treatments for 24 h, 3T3-L1 adipocytes were incubated in 2 mL of prewarmed PBS and 10 M (2 μL) Carboxy-H2DCFDA dye for 30 min at 37 °C. The buffer was subsequently replenished with a prewarmed growth medium, and several fluorescent images were obtained using an inverted immunofluorescent microscope Olympus Microscope BX51 Fluorescence [23,25].

### 2.5. Lipoidperoxidation (MDA) Assay

It is essential to quantify lipid peroxidation to detect oxidative stress in cells, especially adipocytes. The lipid peroxidation malondialdehyde (MDA) assay, therefore, measures lipid peroxidation in the cells according to the manufacturer’s instructions and as published [26]. After hypoxia, normoxia, and DHA treatment for 24 h, the supernatant of the conditioned medium was collected, and the cells were then washed twice with cold PBS. First, cells were homogenized at a density of 5 × 10^5^ cells per sample in an ice-cold MDA Lysis Buffer. Next, the cell lysate was centrifuged, and the supernatant was collected after confirming successful lysis. The samples and standard were then mixed with TBA (thiobarbituric acid) reagent and incubated at 95 degrees Celsius for 60 min, then cooled in an ice bath for 10 min prior to OD 532 nm microplate reader measurements. The data are expressed as nM/g protein adjusted to the number of cells releasing MDA.

### 2.6. Lactate and Glucose Measurements

Evaluation of glucose uptake and lactate generation reveals the anaerobic activity of hypoxic cells. In the MSS (Metabolite Sensitive Sensors) measurement chamber of the Cobas b 123 POC, glucose and lactate were measured by the MSS cartridge, a multiparameter sensor as published [27]. Glucose oxidase and ambient oxygen combine to generate gluconolactone from glucose. Lactate oxidase oxidizes lactate to produce pyruvate. The H_2_O_2_ produced by these reactions is measured amperometrically at 350mV using manganese dioxide/carbon electrodes. The MSS parameters are calibrated using four solutions, and the concentration is used to determine the measured values. Each MSS calibration is compared to a reference measurement using a standby solution. After measuring the calibration solution, this reference measurement is performed. The glucose consumption was measured using the following equation, previously published [28]. Glucose consumption = (A − B)/C. A = glucose concentration in CM (Condition Medium) before treatment. B = glucose concentration in CM post-treatment. C = treatment time (per hour).

### 2.7. Glycerol and Free Fatty Acid (FFA) Release Measurement

All in vitro lipolysis investigations were conducted using 3T3-L1 adipocytes produced and differentiated in 96-well tissue culture plates for 7–10 days after differentiation. 3T3-L1 Adipocytes were equilibrated (0.5–1 h at 37 °C) in DMEM/10% (*v*/*v*) FBS (Fetal Bovine Serum) and then washed twice with 37 °C-prewarmed PBS. To induce lipolysis, PBS was withdrawn and replaced with 0.5 mL of KRH buffer containing 3 percent fatty-acid-free BSA (Invitrogen) and 5 mM glucose. Twenty-four hours of treatment with hypoxia, DHA, and normoxia were carried out at 37 °C in a 5% CO_2_ atmosphere, and the CM was collected for glycerol and FFA assays. Using a lipolysis assay kit, the glycerol and FFA content was assessed per the manufacturer’s recommendations (ZenBio, USA) and previously published [29]. Each test’s optical density is determined at 540 nm using the Tecan microplate reader. Data are reported as M for glycerol or FFA released/g of protein, normalized to cell number.

### 2.8. ATP Measurement 

Following the manufacturer’s directions, 1 × 10^6^ 3T3-L1 cells were lysed, and ATP was measured. The ATP Colorimetric/Fluorometric Assay kit was used (Abcam ab83355) as per the manufacturer’s recommendations and previously published [30]. Fluorometric assays were performed using Perkin Elmer VICTOR 3V Multilabel Plate Readers. Normalized to cell count, ATP was determined as mM/ug protein.

### 2.9. Assessment of Adipocytokines

IL-6, MCP-1, Leptin, and Adiponectin were measured in the conditioned media using enzyme-linked immunosorbent assay (ELISA) kits (Bio Vendor). The assay was accomplished following the manufacturer’s instructions and published elsewhere [31].

### 2.10. Assessment of Quantitative Real-Time RT-PCR

The total RNA was extracted using the TRIzol protocol (Life Technologies, Van Allen Way Carlsbad, CA USA) from treated 3T3L-L1 adipocytes of different groups. Mouse B-Actin primer (Mm00607939 s1) was used as an endogenous control to normalize mRNA expression. The cDNA synthesis from the isolated RNA was performed using a High-Capacity RNA-to-cDNA kit (Applied Biosystems, Foster City, CA, USA). The primer sets are shown in Supplementary Table S2. Quantitative real-time PCR was performed in a reaction containing cDNA and TaqMan Gene Expression Master Mix (Applied Biosystems, Foster City, CA, USA). Samples were analyzed with the Applied Biosystems 7500 Real-Time PCR System (Applied Biosystems, USA). All PCRs were done in triplicate. PCR reaction comprised 52 °C for 1 min, followed by 40 cycles including 90 °C for 30 s, 55 °C for 50 s, and 72 °C for 35 s. The relative amounts of mRNA levels were quantified relative to the expression of the housekeeping gene of mouse B-Actin using the comparative Ct (2-ΔΔCt) value method as published elsewhere [23]. 

### 2.11. Statistical Analysis

Statistical analysis used GraphPad Prism 8 for Windows (GraphPad Software, San Diego, CA, USA). One-way ANOVA was used to compare 3T3-L1 Adipocyte treatments, followed by Tukey’s post-hoc test. The results represent the mean and SD of three biological replicates with at least three independent replicates. Two-tailed *p*-value is 0.05, which is considered significant.

## 3. Results

### 3.1. A-Cellular Effects

**Figure 1:**
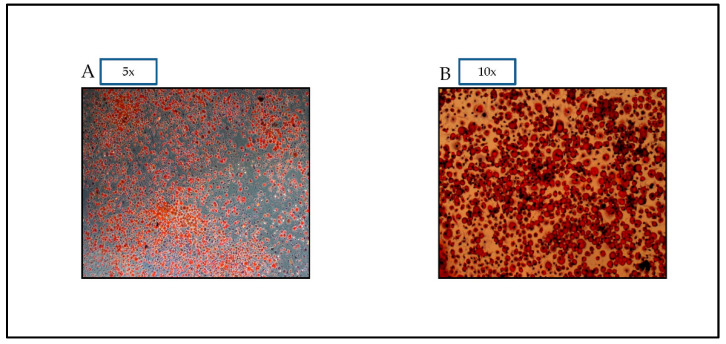
Oil red O pictures of 3T3-L1 cells assessed on Day 7. Oil red O Dye allows for visual confirmation of differentiation. Photographs of stained cells were taken with a microscope OLYMPUS Model DP72 (**A**): magnification 5×, and (**B**): magnification 10×.

Differentiation of preadipocytes to adipocytes is demonstrated by the oil droplets seen in Figure 1. The figure demonstrates that the oil red dye is able to distinguish the differentiated adipocytes from preadipocytes based on the visual appearance of fat droplets.

#### 3.1.1. DHA Treatment Reduces the Hypoxic Effect on Apoptosis and Necrosis in 3T3-L1 Cells

**Figure 2:**
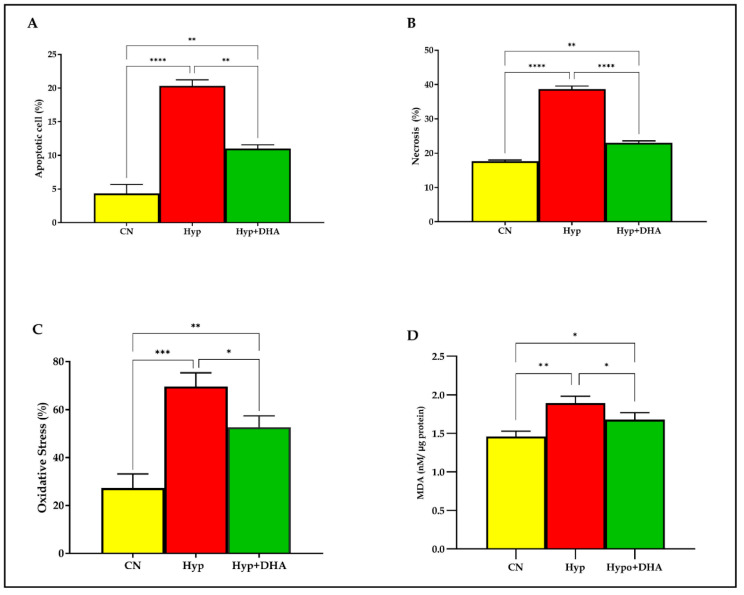
Quantification of apoptosis, necrosis, and oxidative stress. 3T3-L1 adipocytes (7–10 days post-differentiation) were subjected to normoxia, hypoxia (1% O_2_), and hypoxia (1% O_2_) supplemented with DHA (50 nM) for 24 h. They were analyzed for apoptosis in percentages (**A**), necrosis in percentages (**B**), ROS in percentages (**C**), and lipid peroxidation expressed as levels of MDA concentration (nM/ug protein) (**D**). Bars show the data presented as means ±SD of 3–4 independent experiments analyzed by one-way ANOVA and posthoc multiple comparison Tukey tests. Two-tailed *p* value is significant for *p* < 0.05. * *p* ≤ 0.05, ** *p* ≤ 0.005, *** *p* ≤ 0.0005, **** *p* < 0.0001; ns = not significant.

To evaluate the apoptosis and necrosis percentage of 3T3-L1 cells in response to hypoxia and treatment by DHA, Tali® Image-Based Cytometer was used. In short, 3T3-L1 cells exposed to normoxia, hypoxia, and treatment by DHA were stained with green Annexin V-Alexa Fluor® 488. On the other hand, the necrotic cells were stained with red propidium iodide and green Annexin V–Alexa Fluor® 488 and did not stain live cells. The results showed that apoptosis and necrosis of the adipocytes were significantly increased in cells treated with hypoxia by 5 fold and 2.2 fold, compared with normoxia with (*p* ≤ 0.0001) and (*p* ≤ 0.0001) ), respectively. On the other hand, DHA treatment significantly reduced the apoptosis by 1.85 fold and the necrosis by 1.68 fold in the hypoxic cells with (*p* = 0.011) and (*p* ≤ 0.0001), respectively, as shown in Figure 2A,B. 

#### 3.1.2. DHA Treatment Reduces the Hypoxic Effect of Oxidative Stress (ROS) in 3T3L-1 Adipocytes

Oxidative stress determined by the products of the (reactive oxygen species; ROS) in percentages was determined quantitatively by the cell-rox dye (Invitrogen) using Tali image-based cytometer. Exposure to hypoxia significantly increased the total ROS production in 3T3L-1 treated cells by 2.54 fold compared to those exposed to normoxia (*p* < 0.0001). However, DHA treatment significantly reduced the ROS production by 1.32 fold in the hypoxic cells with (*p* = 0.024), as shown in Figure 2C.

#### 3.1.3. DHA Treatment of Hypoxic cells Reduces Lipid Peroxidation Malondialdehyde (MDA)

Lipid peroxidation is distinguished by the oxidative degradation of unsaturated fatty acids, phospholipids, glycolipids, cholesterol esters, and cholesterol. Exposure to hypoxic conditions significantly increased the mean and SD level of lipid peroxidation marker MDA by 1.29 fold from (1.46 ± 0.04 nM/µg protein) in normoxic cells to (1.89 ± 0.053 nM/µg protein) in the hypoxic group, *p* value = 0.002, as shown in Figure 1F. Application of DHA to hypoxic 3T3L-1 adipocytes significantly reduced the MDA by 1.13 fold to (1.77 ± 0.052 nM/µg protein) compared to hypoxic cells (1.89 ± 0.053 nM/µg protein) with *p* = 0.048 as shown in Figure 2D.

#### 3.1.4. Impact of DHA Treatment on H2-DCFDA to Assess ROS 

**Figure 3:**
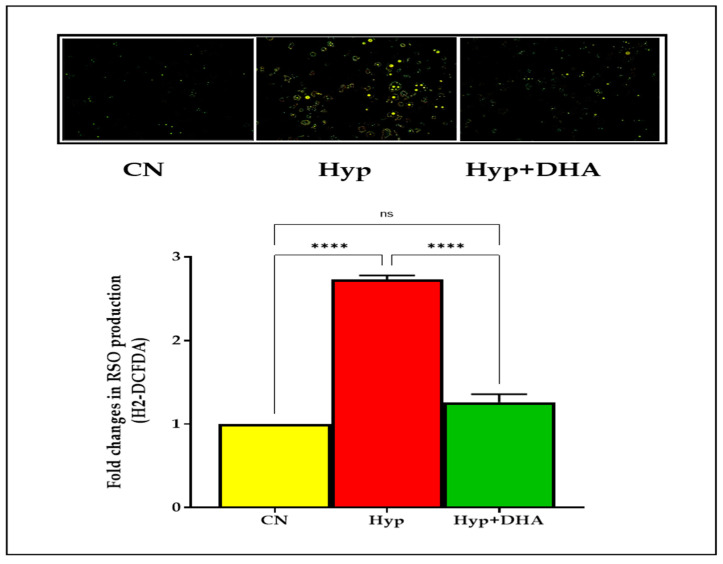
Photograph of ROS immunofluorescence spots expression using carboxy-H2DCFDA dye. 3T3-L1 adipocytes (7–10 days post-differentiation) were subjected to normoxia, hypoxia (1% O_2_), and hypoxia (1% O_2_) supplemented with DHA (50 nM) for 24 h. They were analyzed for ROS production determined by the immunofluorescence of H2DCFDA dye; see Methods section. Data represent 3–5 independent experiments. Bars show the data presented as means ± SD of 3–4 independent experiments analyzed by one-way ANOVA and posthoc multiple comparison Tukey tests. Two-tailed p-value is significant for *p* < 0.05. **** *p* < 0.0001; ns = not significant.

Using a fluorogenic dye H2-DCFDA, the immunofluorescence microscope results demonstrated a significant reduction of ROS after treating hypoxic 3T3L-1 adipocytes with DHA. On the other hand, hypoxia significantly increased the ROS production of normoxic 3T3L-1 adipocyte cells after hypoxia exposure, as shown in Figure 2. In addition, the results showed a marked increase in ROS spots in 3T3L-1 adipocytes exposed to hypoxia compared with the other two groups, as shown in Figure 3.

### 3.2. Metabolic Effects: Impact of DHA Treatment of Hypoxic 3T3L-1 Adipocytes 

#### 3.2.1. Free Fatty Acid and Glycerol in CM

**Figure 4:**
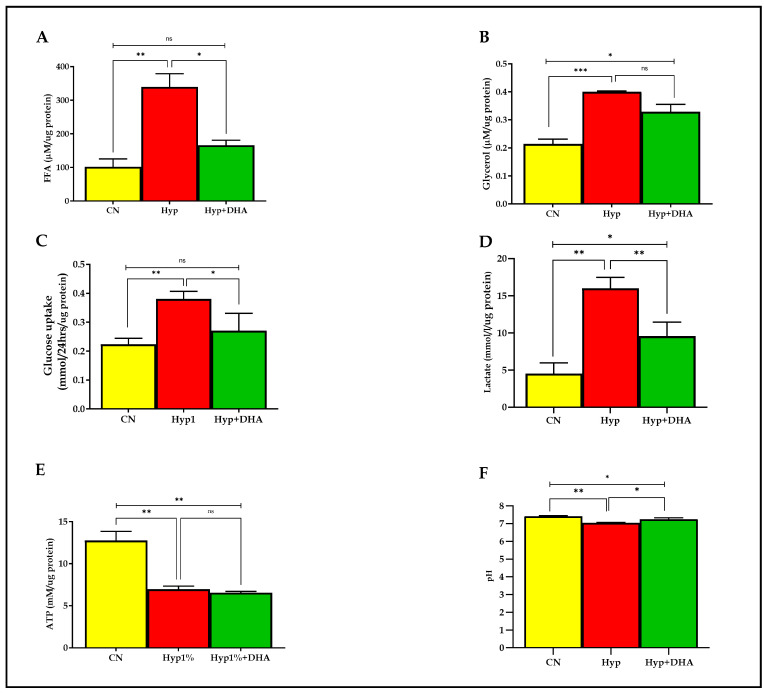
Metabolic effects: impact of DHA treatment on hypoxic 3T3L-1 adipocytes. 3T3-L1 adipocytes (7–10 days post-differentiation) were subjected to normoxia, hypoxia (1% O_2_), and hypoxia (1% O_2_) supplemented with DHA (50 nM) for 24 h. In addition, they were analyzed for FFA release (µM/µg protein) in CM (**A**), glycerol release(µM/µg protein) in CM (**B**), glucose consumption (mmol/24 h/µg protein) in CM (**C**), lactate release (mmol/24h/µg protein) in CM (**D**), and cellular ATP concentrations mM/µg protein (**E**), and pH level in CM (**F**). Bars show the data presented as means ± SD of 3–4 independent experiments analyzed by one-way ANOVA and posthoc multiple comparison Tukey tests. Two-tailed p value is significant if *p* < 0.05. * *p* ≤ 0.05, ** *p* ≤ 0.005, *** *p* ≤ 0.0005; ns = not significant.

Lipolysis was evaluated with free fatty acid and glycerol release into the culture medium of 3T3-L1 adipocytes after exposure to hypoxia and normoxia for 24 h, as detailed in the Methods section. As shown in Figure 4A,B, the free fatty acids and glycerol release were significantly increased in the 3T3-L1 adipocytes treated with hypoxia 1% O_2_ by 3.3 fold (339.3 ± 39.40 µM/µg protein) and 1.9 fold (0.400 ± 0.005 µM/µg protein), respectively, compared with the normoxic cells (101.8 ± 23.34 µM/µg protein) and (0.214 ± 0.029 µM/µg protein), with *p* values of 0.002, and 0.001, respectively. Conversely, the free fatty acids release was significantly decreased in the hypoxic cells treated with DHA by 52% (165.8 ± 25.82 µM/µg protein), with *p* value = 0.011. In contrast, DHA-treated hypoxic cells caused an insignificant decrease of glycerol release in CM, of 18% (0.329 ± 0.027 µM/µg protein), *p* = 0.076. 

#### 3.2.2. Glucose Consumption and Lactate Concentrations in CM

As shown in Figure 4C,D, the mean ± SD of the glucose consumption was significantly higher in the hypoxic group (0.380 ± 0.065 mmol/24 h/µg protein) than in the control group (0.223 ± 0.01 mmol/24 h/µg protein), and *p* value = 0.007, which reflects enhancement of uptake by 173%. DHA treatment of hypoxic cells significantly reduced glucose consumption by 29% (0.270 ± 0.035 mmol/24 h/µg protein) compared with hypoxic cells (0.380 ± 0.065 mmol/24 h/µg protein) with *p* = 0.035, as shown in Figure 4. Of note, no significant change was observed between DHA treatment of hypoxic cells versus the normoxic cells (*p* = 0.388). However, lactate release was significantly increased in the 3T3-L1 adipocytes (7–10 days post-differentiation) treated with hypoxia 1% O_2_ for 24 h by 360% (16.0 ± 0.80 mmol/L/µg protein) compared with untreated normoxic cells (4.53 ± 0.85 mmol/L/µg protein), with *p* value = 0.0003. DHA treatment of hypoxic cells significantly reduced the lactate release (9.57 ± 1.08 mmol/L/µg protein) by 40% in the hypoxic cells (*p* = 0.009), as shown in Figure 4D.

#### 3.2.3. Cellular ATP Level

ATP was quantified from cell lysate in all groups after hypoxia with and without DHA and normoxia conditions described previously in the methodology. As shown in Figure 4E, the ATP concentration (mean ± SD) was significantly lower in the hypoxic group (6.97 ± 0.63 mM/µg protein) than in the controls (12.76 ± 1.88 mM/µg protein), with *p* = 0.002. However, DHA treatment in hypoxic cells showed no significant effect on ATP content (6.55 ± 0.27 mM/µg protein) compared to hypoxic cells, *p* = 0.898. Notably, a significant difference is detected between CN and Hyp+DHA groups with *p* = 0.001.

#### 3.2.4. pH Level in the Conditioned Medium after Hypoxia and DHA Treatment

Further, we evaluated the pH level in CM media before and after 24 h of normoxia, hypoxia, and DHA treatment of hypoxic cells, as shown in Figure 4F. The results showed a significant decrease in the pH of the medium towards acidity with the 3T3-L1 adipocytes exposed to hypoxia (7.034 ± 0.021) compared to 3T3-L1 adipocytes exposed to normoxia (7.410 ± 0.025), with p value of 0.035. Conversely, DHA treatment of hypoxic 3T3-L1 adipocytes significantly increased the pH (to 7.238 ± 0.049) compared with the hypoxic group (*p* = 0.018).

### 3.3. Adipokine Release: Impact of DHA Treatment of Hypoxic 3T3L-1 Adipocytes 

**Figure 5:**
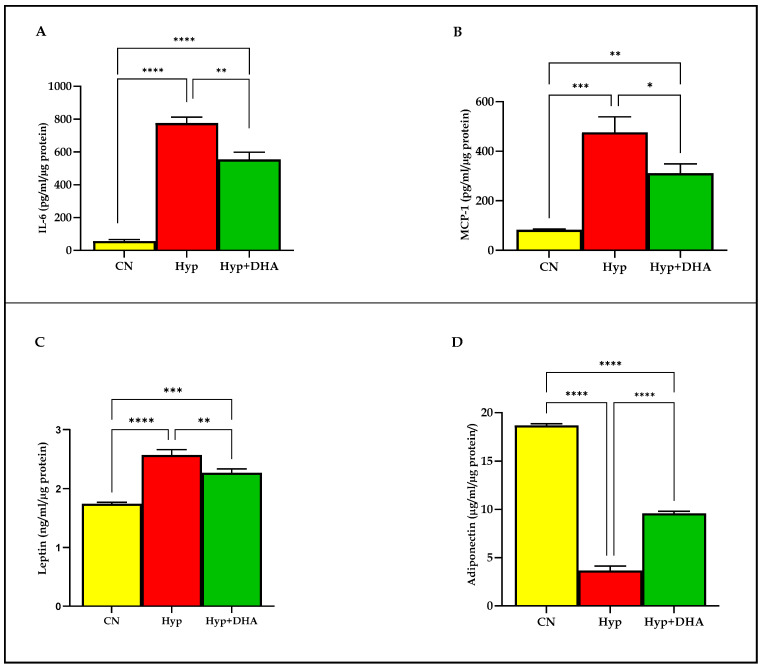
Quantification of adipocytokine release in CM using Eliza: impact of DHA treatment of hypoxic 3T3L-1 adipocytes. 3T3-L1 adipocytes (7–10 days post-differentiation) were subjected to normoxia, hypoxia (1% O_2_), and hypoxia (1% O_2_) supplemented with DHA (50 nM) for 24 h. In addition, they were analyzed by Eliza for Il-6 secretion (pg/mL/µg protein) in CM (**A**), MCP-1 secretion (pg/mL/µg protein) in CM (**B**), leptin secretion (ng/mL/µg protein) in CM (**C**), and adiponectin secretion (µg/mL/µg protein) in CM (**D**). Bars show the data presented as means ± SD of 3–4 independent experiments analyzed by one-way ANOVA and posthoc multiple comparison Tukey tests. Two-tailed *p*-value is significant if *p* < 0.05. * *p* ≤ 0.05, ** *p* ≤ 0.005, *** *p* ≤ 0.0005, **** *p* < 0.0001; ns = not significant.

We examined the release of distinct adipokines from 3T3-L1 adipocytes after 24-h exposure to hypoxia and normoxia. The results demonstrated that hypoxia 1% O_2_ significantly increased the secretion of the following; Il-6 (775.80 ± 36.45 pg/mL/µg protein) compared to the normoxic cells (56.76 ± 8.92 pg/mL/µg protein), *p* ≤ 0.0001 (Figure 5A); MCP-1 (83.07 ± 1.69 pg/mL/µg protein) compared to the normoxic cells (476.20 ± 36.25 pg/mL/µg protein), *p* = 0.0002 (Figure 5B); leptin (2.57 ± 0.05 ng/mL/µg protein) compared to the normoxic cells (1.74 ± 0.015 ng/mL/µg protein), *p* < 0.0001 (Figure 5C). In contrast, hypoxia significantly decreased the release of adiponectin from 3T3-L1 adipocytes (3.66 ± 0.26 µg/mL/µg protein) compared to the normoxic cells (18.69 ± 0.09 µg/mL/µg protein), *p* < 0.0001 (Figure 5D). 

DHA treatment of hypoxic cells significantly decreased the secretion of: Il-6 (554.50 ± 44.40 pg/mL) compared to hypoxia 1% O_2_ (775.80 ± 36.45 pg/mL/µg protein), *p* = 0.008 (Figure 5A); MCP-1 (311.20 ± 37.66 pg/mL) compared to hypoxia 1% O_2_ (476.20 ± 36.25 pg/mL/µg protein), *p* = 0.019; leptin (2.27 ± 0.03 ng/mL/µg protein) compared to hypoxia 1% O_2_ (2.57 ± 0.05 ng/mL), *p* = 0.003 (Figure 5C). In contrast, hypoxia 1% O_2_ +DHA significantly increased the release of adiponectin from 3T3-L1 adipocytes (9.58 ± 0.12 µg/mL/µg protein) compared to the hypoxic cells (3.66 ± 0.26 µg/mL/µg protein), *p* < 0.0001 (Figure 5D).

### 3.4. Gene Expression: Impact of DHA Treatment on Hypoxic 3T3L-1 Adipocytes 

The expression of hypoxia-responsive genes was tested by qPCR to define the hypoxia response. The genes include HIF1α, HIF2α, GLUT1, GLUT4, Ladh, PPARγ, FASN, and ANT2.

**Figure 6:**
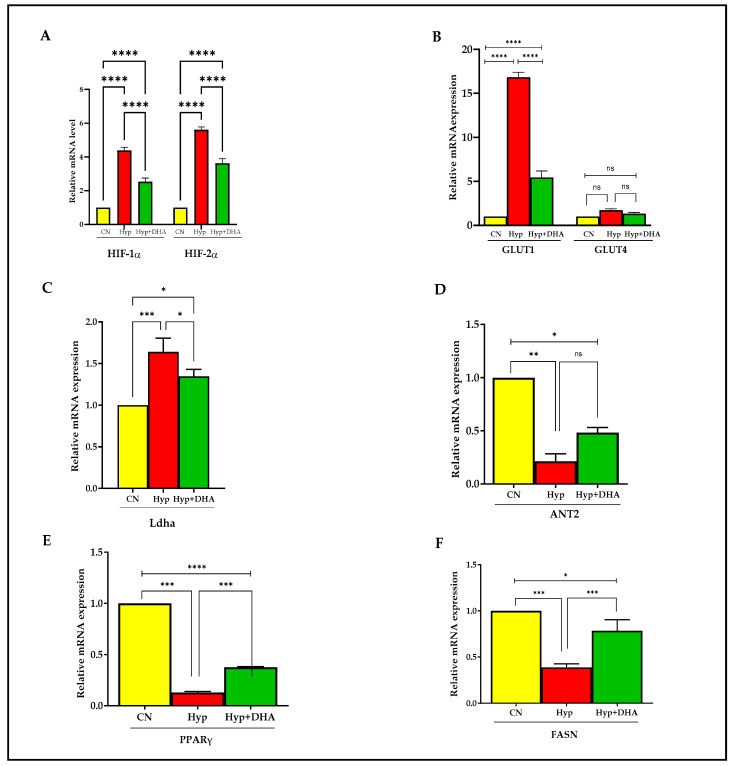
Quantification of mRNA level expression in 3T3L-1 adipocytes in response to hypoxic and DHA by RT-PCR. (**A**–**F**): Hypoxia-induced gene expression (mRNA) in normoxia, hypoxia, and hypoxia + DHA (50uM) treatment in 3T3-L1 cells was determined against β-actin as a housekeeping gene by RT-PCR. Panel (**A**): HIF-1α, HIF-2α; panel (**B**): GLUT1 and GLUT4; Panel (**C**): Ladh; Panel (**D**): ANT2; Panel (**E**): PPARy; Panel (**F**): FASN. Bars show the data (mRNA) presented as means ± SD of 3 independent experiments analyzed by one-way ANOVA and posthoc multiple comparison Tukey tests. Two-tailed p value is significant if *p* < 0.05. * *p* ≤ 0.05, ** *p* ≤ 0.005, *** *p* ≤ 0.0005, **** *p* < 0.0001; ns = not significant.

Hypoxia-inducible factor 1, alpha subunit (Hif1a), and hypoxia-inducible transcription factor 2alpha (HIF2α) expression increased significantly in 3T3L-1 treated with hypoxia by ≈ 4.3 fold and 5.1 fold compared to cells exposed to normoxia, with *p* value < 0.0001 and *p* < 0.0001, respectively (Figure 6A). In comparison, HIF1α and HIF2α expression decreased significantly in hypoxic cells treated with DHA by 40% and 35%, respectively, compared with hypoxic cells; *p* < 0.0001 for both genes (Figure 6A). 

Hypoxia 1% caused significant upregulation of solute carrier family 2 (Slc2a1) facilitated glucose transporter, member 1, known as GLUT1, by 15 fold, *p* < 0.0001, and DHA treatment of hypoxic cells caused significant downregulation by 67% compared with hypoxic cells, *p* < 0.0001 (Figure 6B). On the other hand, solute carrier family 2 (Slc2a4) facilitated glucose transporter, member 4, known as GLUT4 expression, increased insignificantly in 3T3L-1 treated with hypoxia by 1.7 fold compared to cells exposed to normoxic cells, with *p* = 0.231, and further treatment by DHA to hypoxic cells insignificantly downregulated the expression by 20%, with *p* = 0.606 (Figure 6B).

Hypoxia caused significant upregulation of lactate dehydrogenase A (Ladh) expression by 1.7 fold, with *p* = 0.008, and DHA treatment of hypoxic cells caused significant downregulation of Dhla expression by 20% compared with hypoxic cells, *p* = 0.034 (Figure 6C).

Furthermore, hypoxia caused significant downregulation of expression by ADP/ATP translocase 2, known as (Slc25a5 or ANT2), by 4.7 fold, with *p* = 0.003, and DHA treatment of hypoxic cells caused insignificant upregulation of Dhla expression by 22% compared with hypoxic cells, *p* = 0.061 (Figure 6D).

Exposure to 3T3L-1 treated with hypoxia caused significant downregulation of Peroxisome proliferator-activated receptor gamma (PPARγ) expression, by 87%, with *p* < 0.0001. Conversely, DHA treatment of hypoxic cells caused significant upregulation of PPARγ expression by 285% compared with hypoxic cells, *p* < 0.0001 (Figure 6E). 

Fatty acid synthase (FASN) is a multifunctional enzyme involved in fatty acid biosynthesis. Treatment of 3T3L-1 cells with hypoxia caused a significant downregulation of FASN expression by 62%, *p* < 0.0001. Conversely, DHA treatment of 3T3L-1 cells exposed to hypoxia caused significant upregulation of FASN expression by 200% compared with hypoxic cells, *p* = 0.001 (Figure 6F). 

## 4. Discussion

Hypoxia is one of the primary contributors to the dysfunctions of adipocytes in obesity. In numerous organs, oxygen plays a crucial role in the metabolic control of carbohydrates and lipids [32]. The oxygen tension within the human body is lower than in the surrounding atmosphere and varies between 0.5 and 14.0 percent in different tissues [33]. In the present work, we assessed the effect of docosahexaenoic acid (DHA) polyunsaturated fatty acid on adipocyte dysfunctions and whether it could mitigate hypoxic effects. Therefore, we examined the effect of DHA therapy on murine 3T3-L1 adipocytes in a hypoxic condition (1% O_2_), which replicates the AT microenvironment in obese individuals. We analyzed the oxidative stress, metabolic substrates, ATP content, and adipocytokines in response to hypoxia and their changes following DHA application.

Exposure of adult adipocytes to hypoxia (1% O_2_) for 24 h drastically altered the metabolism from aerobic to anaerobic with changes in metabolic substrates of glucose and lipid metabolism, as demonstrated by the present study. However, DHA therapy of hypoxic cells reduced these unfavorable consequences by counteracting the effects of hypoxia on metabolism, gene expression related to metabolic alterations, apoptosis, pro-inflammatory responses promoting ROS generation, antioxidant enzymes, and adipokine secretion. In the following paragraphs, these results and their ramifications are further considered.

3T3-L1 mouse adipocytes were treated with (1% O_2_) for 24 h in the current investigation to induce hypoxia, which corresponds to 7.6 mm Hg of PO_2_, comparable to the level reported in the WAT of the obese mice (ob/ob) [34]. The pathophysiology of obesity is based on adipose tissue dysfunction characterized by adipocyte hypertrophy, decreased adipokine release, chronic low-grade inflammation, hormonal resistance, and altered metabolic responses, predisposing to insulin resistance [35]. Therefore, due to decreased adipocyte oxygenation, local hypoxia in obese individuals may contribute to AT dysfunction.

Hypoxia stimulated the HIF-1α gene, which in turn altered the expression of nearly 70 genes [36]. HIF-1α activated gene is mainly associated with oxidative stress, inflammation, glucose metabolism, angiogenesis, and apoptosis [18]. In low-oxygen settings, the principal purpose of these genes is to promote cell survival by promoting angiogenesis and glycolytic metabolism [37]. In addition, stimulation of transcript elements such as HIF1 plays a crucial role in hypoxia-induced stimulation of inflammatory chemokine and cytokine production, including leptin, adiponectin, IL-6, VEGF, PAI-1, and MCP-1 [38]. These cytokines probably have a role in macrophage infiltration into adipose tissue [39,40]. In the current study, exposure of 3T3-L1 murine adipocytes to hypoxia resulted in the upregulation of HIF-1α and HIF-2α in response to hypoxia, which was mitigated after DHA application. The result of the current study agrees with previous studies demonstrating upregulation under hypoxic conditions [6,41,42]. HIF-1α is extensively expressed in mammalian tissues, while HIF-2 is expressed in specific tissues, including blood vessels, kidneys, liver, pancreas, heart, lungs, gut, and brain. HIF1 and HIF2 are transcriptional activators regulating multiple biological processes, including glucose and lipid metabolism, angiogenesis, mitochondrial function, and inflammation. Under normoxic conditions, prolyl hydroxylase enzymes (PHDs) rapidly degrade the intracellular HIF-1α, which is unstable. In addition, these enzymes are inhibited by low oxygen levels, suggesting that hypoxia does promote HIF-1α. In addition to hypoxia, reactive oxygen species (ROS), nitric oxide (NO), metabolic intermediates of the tricarboxylic acid (TCA) cycle, proinflammatory cytokines such as tumor necrosis factor-(TNF-α), interleukin-1 (IL-1), and hormonal factors play a central role in the control of HIF stabilization and transcription activity, respectively [43]. The recent study’s data showed the downregulation of HIF1α and HIF2α expression in response to DHA application to hypoxic cells. A recent fish oil study showed that docosahexaenoic acid (DHA) significantly reduced HIF-1α expression caused by maternal hypoxia in pregnant rats [44]. A prior study reported a significant decrease in HIF-1α expression following a clinical intervention trial of n3 PUFA therapy in subcutaneous abdominal biopsies from highly obese, nondiabetic patients slated to undergo elective bariatric surgery [45]. These investigations complement our findings on the downregulation of HIF-1a expression in hypoxic cells following DHA therapy. According to our data, possible mechanisms of the downregulation of HIF-1α and HIF-2α may be secondary effects of DHA administration which reduces oxidative stress, inflammation, and apoptosis. These mechanisms have been mentioned as potential biological processes through which DHA regulates HIF stability and transcriptional factors and are further explored in the following sections [43]

### 4.1. DHA’s Anti-Apoptotic Activity Mitigates Hypoxia-Induced Apoptosis

The current study disclosed that exposure of mature 3T3L1 cells to hypoxia causes an increased rate of apoptosis and necrosis, which is ameliorated by DHA treatment. ATP generation in mitochondria is controlled by hypoxia. Therefore, hypoxia is suspected of being the cause of necrosis and apoptosis due to its effect on ATP production. The increased apoptosis rate and basal lipolysis triggered by hypoxia result in a decrease in glucose metabolism and a shift to anaerobic metabolism. Numerous mechanisms of apoptosis and necrosis triggered by hypoxia have been identified in previous research. First, hypoxia can trigger apoptosis by inducing hyperpermeability of the inner mitochondrial membrane, releasing cytochrome C, which slows the transport of protons, hence generating a drop in membrane potential and energy deprivation, promoting apoptosis [46]. Second, hypoxia resulted in a considerable drop in adenine nucleotide translocator (ANT2) mRNA expression, as demonstrated by the present study’s findings. ANT2 plays a vital function in ATP generation by preserving mitochondrial integrity and avoiding oxidative phosphorylation-related protein changes [47]. Another mechanism by which hypoxia triggers apoptosis is radical production, namely reactive oxygen species (ROS) generation, when initiator caspase 9 is cleaved directly to the active form by caspases 3 and 12 without the involvement of cytochrome C [48]. The results of this investigation indicated an increase in reactive oxygen species (ROS) production and lipid peroxidation (MAD), which may contribute to the hypoxia-induced apoptosis seen in this study. Cells may shift from apoptosis to necrosis depending on the severity of hypoxia and lack of energy deprivation. Previous reports demonstrated that DHA could induce apoptosis and inhibit cell proliferation in cells such as cancer cells [49]. In addition, a previous study reported that DHA inhibits adipocyte differentiation by inducing apoptosis in 3T3-L1 post-confluent pre-adipocytes after 48 h of treatment with 200 M/L of DHA, which could be a mechanism of anti-adipogenesis and fewer adipocyte numbers [50].

Furthermore, Kim et al. reported that DHA had been shown to increase basal lipolysis in fully differentiated normal 3T3-L1 adipocytes under normoxic conditions, which contradicts our findings in the present study under hypoxic conditions. Therefore, we cannot compare our findings on DHA supplementation to hypoxic cells as we provided DHA for 24 h with a dose of 50 μM (lower dose) compared to Kim et al.’ study, and also apoptosis was induced by severe hypoxia of low oxygen tension 1% in our study. However, in summary, our data could refer to a protective effect of DHA against the deleterious effects of hypoxia on the metabolic energy system, as evidenced by increasing ATP levels, decreasing lactate production, and shifting metabolism from anaerobic to aerobic with the reduction of apoptotic rate. Therefore, it is proposed that DHA preserves energy stores to counteract hypoxic effects on adipocytes in such abnormal conditions.

### 4.2. Impact of DHA on Oxidative Stress and Lipid Peroxidation

It is well-established that obesity is associated with oxidative stress, which leads to various comorbidities, including insulin resistance, cardiovascular difficulties, and endothelial dysfunction [51]. The present study data demonstrated that the DHA supplement reduces the ROS generated by hypoxia. Furthermore, DHA application ameliorates the lipid peroxidation (MAD) produced in response to hypoxia. Previous studies indicated that exposure to low O_2_ tension increases adipocyte ROS production, including by 3T3-L1 cells [52]. In addition, higher ROS levels in response to hypoxia play a role in stabilizing HIF-1, which is essential for its function as the primary regulator of cellular responses to low O_2_ availability [53]. Excessive ROS production can cause a negative impact, including DNA damage and lipid peroxidation. The accumulation of excessive fat in WAT induced an increase in lipid peroxidation in WAT itself, accompanied by a rise in NOX activity and a decrease in the mRNA expression and activities of antioxidant enzymes such as SOD, Catalase (CAT), and GPx in WAT [54]. DHA attenuates the increase of total ROS production and lipid peroxidation in mature 3T3L1 adipocytes. The significance of such changes observed in response to DHA could reduce the damaging effect of excessive ROS caused by severe hypoxia and prevent lipid peroxidation and damage.

Furthermore, decreasing oxidative stress and ROS are linked to apoptosis and inflammatory profile changes, and metabolic changes of hypoxic adipocytes were observed after DHA application. In a previous study using lipopolysaccharide-induced inflammation, DHA protected Atlantic salmon from oxidative stress and prevented fat deposition, as shown by the increased SOD2 activity and transcriptional modulation of antioxidant enzymes and pro-and anti-inflammatory markers [55].

Furthermore, in a previous study by Kusunoki et al., 3T3-L1 adipocytes were treated with varying doses of (DHA) for different durations, and it was shown that DHA has an anti-oxidative impact by raising the production of HO-1 via the activation of the Nrf-2 pathway and inhibits cytotoxicity generated by H_2_O_2_ an HO-1-dependent manner [56]. 

### 4.3. Anti-Inflammatory Effects of DHA Supplementation following Hypoxia

Several experiments have demonstrated that hypoxia-inducible factor 1 (HIF-1) signaling plays a vital role in inflammation and insulin resistance associated with obesity [36].

The present study demonstrated that hypoxia increased the pro-inflammatory mediators IL-6, MCP-1, leptin secretion, and NFKb mRNA expression in 3T3-L1 adipocytes as reported in human adipocytes [57], whereas 24 h of DHA treatment suppressed these effects. These results are consistent with prior research demonstrating increased macrophage chemotactic protein (MCP)-1 secretion in the condition media obtained from adipocytes cultured in 1% O_2_ [58]. Il-6 secretion decreased in CM after exposure of adipocytes to hypoxia, as previously published [57]. IL-6 is an acute-phase response inducer in the context of elevated stress levels and inflammation or infectious disease. Increased IL-6 release from adipose tissue contributes to the development of insulin resistance either by reducing adiponectin secretion, as demonstrated by our findings, or by influencing the insulin signal transduction pathway in hepatocytes [59]. The contribution of MCP-1/CCR2 in obesity-induced inflammation revealed increased gene expression of CC chemokines and their receptors (such as MCP-1 and CCR2) in visceral and subcutaneous adipose tissues of obese patients compared to lean subjects [60]. MCP-1 deficiency is associated with macrophage accumulation in adipose tissue and insulin sensitivity, so it is a fundamental biomarker of leukocyte recruitment to the site of the inflammation [61]. DHA was the most potent anti-inflammatory agent, decreasing MCP1 and IL-6 secretion in co-culture 3T3L1 with RAW 264.7 macrophages [62].

In addition, the current work demonstrates that DHA treatment reduces the enhanced leptin release in hypoxia-exposed adipocytes. In accordance with our findings, leptin is a hypoxia response gene whose transcription is stimulated by HIF-1 [63]. Leptin is a hormone that impacts energy balance and body mass. Moreover, hyperleptinemia is involved in insulin resistance, which may clarify why hypoxia could induce insulin resistance by increased secretion of pro-inflammatory cytokines such as Il-6, MCP-I, and leptin with decreased adiponectin, as evidenced in the present study, which is consistent with prior research [64]. DHA reduced leptin mRNA expression in 3T3-L1 adipocytes [65]. In a study by Zebrowska et al., human subjects who received two doses of 267 mg of DHA for three weeks exhibited a significant decrease in circulating leptin concentration and an increase in adiponectin level, which was accompanied by a decrease in plasma TNF and oxidative stress markers and an improvement in lipid profile [66]. Consistent with prior research, our results revealed that 3T3L-1 mature adipocytes exposed to low oxygen tension (1% O_2_) exhibited a substantial drop in adiponectin release [7,67]. As demonstrated by an earlier study, adiponectin secretion is dependent on O_2_ level, with 10% O_2_ increasing adiponectin secretion by 2.2 fold compared to 1% O_2_, and 21% O_2_ increasing adiponectin secretion by 3.6 fold compared to 1% O_2_ [68]. In addition, it is well known that inflammation caused by hypoxia induces TNF-alpha, inhibiting adiponectin mRNA expression in adipocytes [69]. In a prior work by Oster et al., DHA incubation for 24 h boosted adiponectin mRNA expression and protein secretion in 3T3-L1 adipocytes [70]. Gonzalez-Periz et al. demonstrated an increase in adiponectin mRNA levels in adipose tissue of ob/ob mice fed a DHA-rich diet for 5 weeks [71]. Consistent with earlier research, DHA administration to hypoxic cells stimulates adiponectin secretion [20]. Adiponectin expression in adipocytes is enhanced by PPAR-γ [72], which could clarify the increase of adiponectin after DHA treatment, as it upregulated PPAR- γ expression, hence preventing the decrease in adiponectin expression induced by IL-6 in our study. Increased adiponectin secretion may reflect the therapeutic action of DHA in combating the stigma of insulin resistance associated with hypoxia effects on adipocytes and their effect on fat and glucose metabolism. 

### 4.4. DHA Counteracts Hypoxia-Induced Metabolic Alterations in Adipocytes

The current study indicated an increase in baseline glucose consumption and lactate release in 3T3-LI adipocytes exposed to hypoxia for 24 h. A previous work by Yin et al. demonstrated that hypoxia promotes glucose metabolism in adipocytes via insulin-dependent and independent mechanisms [73]. Multiple processes, including the overexpression of particular genes such as hexokinase 2 (HK2), phosphofructokinase (PFKP), and glucose-6 phosphate isomerase (GPI), have been implicated in the hypoxia-induced activation of the glycolytic pathway [74]. Another mechanism is GLUT1 expression, which is crucial for baseline glucose uptake and has been demonstrated to be upregulated under hypoxic conditions in earlier investigations [75]. The GLUT1 transcript was significantly upregulated in 3T3-LI adipocytes subjected to hypoxia, consistent with earlier research [36]. However, the data from the present work revealed that after 24 h of hypoxia, GLUT-4 expression remained unchanged. The insignificant change in GLUT4 expression in the present study could refer to the lack of hypoxic effects on insulin-stimulated glucose transport, as glucose uptake is dependent on GLUT1. In hypoxia, the hexosamine pathway inhibits GLUT-4, which lowers GLUT-4-mediated glucose uptake and, in conjunction with reduced adiponectin, causes insulin resistance. A previous study has revealed that the increase of GLUT4 in 3T3-L1 adipocytes with a HIF-1B deletion may enhance insulin sensitivity and signaling in adipocytes [76].

DHA therapy of hypoxia cells had a negligible effect on GLUT4 expression, indicating possible additional mechanisms involved in reducing insulin resistance in treated hypoxic cells other than glucose uptake via the GLUT4 pathway. These results are consistent with previous studies that reported that DHA could modify the expression of GLUT4 gene, which is connected with insulin sensitivity and glucose metabolism [20]. Therefore, DHA could be linked with insulin resistance, preventing the decrease in GLUT4 [77]. In 3T3-L1 cells, IL-6 induced insulin resistance by inhibition of the gene of GLUT 4 [78]. A significant correlation can be proposed by gathering the results about the effect of DHA on IL-6, MCP1, adiponectin, and leptin genes. Thus, the conclusion is that IL-6 is one of the essential adipokines that directly increase insulin resistance in adipocytes [67].

Consistent with earlier findings, the current study found an increase in lactate levels and a drop in pH in the CM of the adipocytes subjected to hypoxia, as well as an activation of the Ldha gene expression [75]. Inflammation and insulin resistance can be induced in muscles by lactate. Hypoxia and insulin resistance in adipose tissue may increase lactate generation and antilipolytic action [79]. As shown in the present work, the decrease in baseline glucose uptake caused by DHA treatment of hypoxic cells would result in less lactate formation and release.

Moreover, our data demonstrated a significant decrease in the adenine nucleotide translocator (ANT2) mRNA, consistent with a prior study [47]. In our study, DHA in hypoxic conditions reduces the effect of the hypoxia by preventing further decrease and maintaining a stable level of ATP production and ANT2 in 3T3-L1 cells. In normal conditions, mitochondria produce intermediates that are essential for lipogenesis [80]. However, insulin and insulin-like growth factors could inhibit the lipolysis process. A recent study has shown that DHA can protect the mitochondrial shape and function under induced inflammation, and it has anti-inflammatory properties and protects against oxidative stress and lipid accumulation [55]. Thus, modifying ATP and ANT2 production under DHA treatment can help cells to survive under hypoxic conditions.

In addition, enhancing mitochondrial respiration with DHA inhibits the anaerobic route, resulting in a minor lactate release. A previous study suggested that lactate produced from glucose mediates the antilipolytic effect of insulin in white adipose tissue through an autocrine/paracrine loop [81]. The current data demonstrated an increase in lipolytic activity as measured by the release of glycerol and free fatty acids from 3T3L1 adipocytes subjected to hypoxia at the basal state. These results are similar to a prior study that demonstrated increased lipolysis in 3T3-L1 adipocytes following treatment with hypoxia (1% O_2_) [56]. Hypoxia decreased the expression of the PPAR gene, as demonstrated by our data, which is in line with previous findings [82], and the enhanced basal lipolysis and lower fat synthesis in 3T3-L1 adipocytes may be explained by the downregulation of the FASN gene, which is a marker of fat synthesis. The deleterious insulin action on adipocytes may be the underlying cause of hypoxia-induced lipolysis. The hypoxia-induced downregulation of PPAR gene expression has been hypothesized as a mechanism for enhanced lipolysis [82]. PPAR-γ is a transcription factor primarily expressed in adipose tissue, and its activation, as observed in hypoxic adipocytes exposed to DHA, is believed to reduce insulin resistance [72]. The elevated levels of circulating FFA represent a causal factor in developing insulin resistance [83]. DHA treatment of hypoxic cells significantly attenuated FFA release without significantly impacting glycerol release. These changes could refer to the inhibition of lipolysis without corresponding alterations in triglyceride accumulation (no change in glycerol release). As a result of these changes brought about by DHA administration during hypoxia, the release of FFA is restricted, insulin resistance is counteracted as indicated by increased PPARy expression, and adipocyte metabolism improves, hence limiting fat formation as indicated by decreased FASN expression, and consequently adipocyte enlargement (hypertrophic obesity). DHA promotes fatty acid oxidation via activating AMP-activated protein kinase (AMPK) in 3T3-L1 adipocytes, according to earlier studies [84]. Lipolysis in adipocytes has been found to promote the production of inflammatory cytokines, including Il-6 and MCP-1. These proinflammatory mediators decrease the generation of adiponectin, which is controlled by PPARy inactivation, resulting in insulin resistance. Consequently, increased oxidative stress and lipid peroxidation could be reversed by DHA administration as a result of insulin resistance, according to the present study [38,85].

In summary, this work investigates the anti-hypoxia effects of DHA treatment on 3T3-l1 adipocytes. In the current study, the administration of DHA to hypoxic cells reduced oxidative stress, apoptosis, the production of pro-inflammatory cytokines, and leptin and upregulated adiponectin secretion through the stimulation of PPAR gamma. In addition, DHA inhibits the HIF1-alpha gene and consequently alters the expression of numerous genes involved in inflammation (IL6, MCP-1, leptin), transcription factors (NFkB), metabolism (GLUT1, Ldha, FASN, ANT2), and the transition from anaerobic to aerobic metabolism with inhibition of FFA release. Furthermore, due to the increase of PPAR-Y signaling and suppression of NFkB signaling, DHA may indirectly enhance insulin sensitivity produced by hypoxia.

This research had several drawbacks, including that only one level of hypoxia was used (1% oxygen), the hypoxia treatment was continuous, and a mouse model of adipocytes was not utilized to highlight DHA effects upon hypoxia exposure.

Future studies could address such limitations and include new techniques, such as next-generation sequencing, to detect new biomarkers/signaling pathways using relevant human tissue from different depots and animal models.

## 5. Conclusions

The present study demonstrated that adipocytes exposed to hypoxia altered adipokine secretion, the transcript of hypoxia genes, and other genes involved in glucose and lipid metabolism. Hyoxia is associated with a shift toward anaerobic metabolism with increased oxidative stress and apoptosis. In addition, the study showed the potent effect of DHA in counteracting the hypoxic effects on adipocytes and identified several key factors mediating DHA functioning. The beneficial effects of DHA emerged by eliciting several bioactive molecules in 3T3L-1 under hypoxia conditions. These molecules operate within complex systems such as inflammation factors, lipolysis system, glycolysis process, reactive oxygen species, and various cellular genes involved in adipocyte functioning. Consideration may be given to the potential of DHA as a therapeutic agent to reduce obesity and associated comorbidities.

## Supplementary Materials

The following supporting information can be downloaded at: https://www.mdpi.com/article/10.3390/nu14214600/s1, Figure S1: Cell Line: ZenBIO 3T3-L1 Preadipocytes; Table S1:Description of media formulations for cell differentiation as provided by Zen-BIO; Table S2: Primers.
