# Supplementary material for: Docosahexaenoic Acid Counteracts the Hypoxic-Induced Inflammatory and Metabolic Alterations in 3T3-L1 Adipocytes
Source: Nutrients. 2022 Nov 1;14(21):4600. doi: 10.3390/nu14214600 (PMC9659308; doi:10.3390/nu14214600)

Manuscript title:

Docosahexaenoic acid Counteracts the Hypoxic-Induced Inflammatory and Metabolic Alterations in 3T3-L1 Adipocytes

Table S1. Description of media formulations for cell differentiation as provided by Zen-BIO

MEDIUM	FORMULATION
3T3-L1 Preadipocyte Medium	DMEM, high glucose HEPES pH 7.4 Bovine Calf Serum (BCS) Penicillin Streptomycin Amphotericin B
3T3-L1 Adipocyte Medium	DMEM / Ham's F-12 medium (1:1, v/v) HEPES pH 7.4 Fetal Bovine Serum (FBS) Biotin Pantothenate Human insulin Dexamethasone Penicillin Streptomycin Amphotericin B
3T3-L1Differentiation Medium	DMEM / Ham's F-12 medium (1:1, v/v) HEPES pH 7.4 Fetal Bovine Serum (FBS) Biotin Pantothenate Human insulin Dexamethasone Penicillin Streptomycin Amphotericin B Isobutylmethylxanthine PPARγ agonist

Table S2. Primers

TaqMan Gene Expression Assay (Mouse **PPARγ** primer): [Mm01184322_m1]/cat# 4331182/Applied Biosystems Foster, TheromFischer, USA
TaqMan Gene Expression Assay B-actin (Mouse **Actb** primer): (Mm00607939_s1)cat# 4331182/ Applied Biosystems Foster, TheromFischer, USA. Housekeeping gene.
TaqMan Gene Expression Assay **Slc1a3** (Mouse **GluT-1** primer): [Mm00600697_m1]/cat# 4331182/Applied Biosystems Foster, TheromFischer, USA
TaqMan Gene Expression Assay (Mouse **Hif1a** primer); [Mm00468869_m1]/cat# 4331182/ Applied Biosystems Foster, TheromFischer, USA
TaqMan Gene Expression Assay (Mouse **FASN**- mouse primer): [Mm00662319_m1]/cat# 4331182/Applied Biosystems Foster, TheromFischer, USA
PrimeTime Mini qPCR Assay, Mm.PT.58.13819524, **Hif-2 alpha**<br>Probe 5′-/56-FAM/ACC AGA GCC /ZEN/GTT TTT GAG AGT CAG G/3IABkFQ/-3′<br>Primer1 5′-GAC ACG TCT TTG CTC TTC TTC-3.′<br>Primer2 5′-GAC TTC ACT CAT CCT TGC GA-3.′<br>IDT Integrated DNA Technologies USA
PrimeTime Mini qPCR Assay, Mm.PT.58.9683859, **GLUT 4**<br>Probe, 5′- /56-FAM/TGG AAA CCC /ZEN/GAC GGC ATC TTG T/3IABkFQ/-3′<br>Primer 1 5′-GAG AAT ACA GCT AGG ACC AGT G-3′<br>Primer 2, 5′-TCT TAT TGC AGC GCC TGA G-3.′<br>IDT Integrated DNA Technologies USA
PrimeTime Mini qPCR Assay, Mm.PT.58.32860004, **ANT 2**<br>PrimeTime Probe, 5′-/56-FAM/TCA CGG CAG /ZEN/ATA AGC AAT ACA AGG GC/3IABkFQ/-3.′<br>Primer1,5′-GAT ACG AAC CAC GCA GTC TAT G-3′<br>Primer 2,5′-GCA GCC ATC TCC AAG ACA G-3.′<br>IDT Integrated DNA Technologies USA

Figure S1. Cell Line: ZenBIO 3T3-L1 Preadipocytes

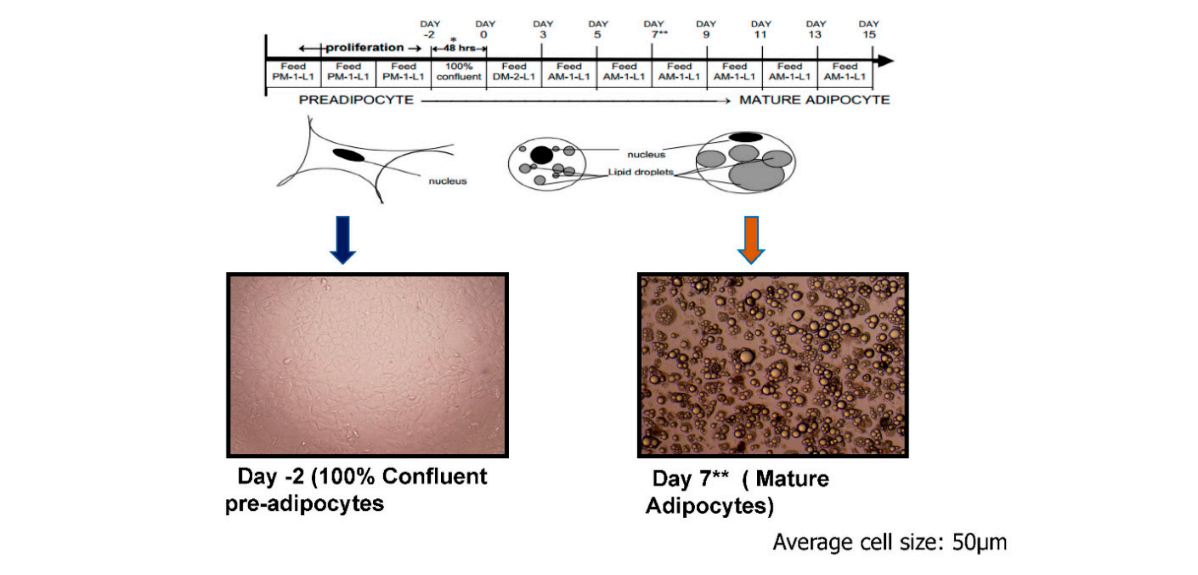

Supplement: Supplementary file 1 [file nutrients-14-04600-s001.zip › nutrients-1930683-supplementary.pdf]
